# Residual Dynamics of Fluopyram and Its Compound Formulations in *Pinus massoniana* and Their Efficacy in Preventing Pine Wilt Disease

**DOI:** 10.3390/plants15020302

**Published:** 2026-01-20

**Authors:** Wanjun Zhang, Anshun Ni, Jiao Zhang, Guohong Sun, Fan Xiang, Hao Cheng, Tingting Chen, Jianren Ye

**Affiliations:** 1 Co-Innovation Center for Sustainable Forestry in Southern China, College of Forestry, Nanjing Forestry University, Nanjing 210037, China; zwjnjfu@njfu.edu.cn (W.Z.); jiaoooz@163.com (J.Z.); sunguohong@njfu.edu.cn (G.S.); m18036964817_1@163.com (F.X.); ch248375@njfu.edu.cn (H.C.); ttchen@njfu.edu.cn (T.C.); 2 Jiangsu Key Laboratory for Prevention and Management of Invasive Species, Nanjing Forestry University, Nanjing 210037, China; anshunni@163.com; 3 Forestry Station of Chongming District, Shanghai 202150, China

**Keywords:** *Bursaphelenchus xylophilus*, trunk injection, agent residues, disease prevention effect

## Abstract

Injecting chemical agents into tree trunks is a key method for preventing pine wilt disease (PWD). However, the long-term use of conventional trunk injection agents such as emamectin benzoate (EB) and avermectin (AVM) may lead to nematode resistance. Therefore, it is crucial to evaluate the potential of new-generation nematicides, including fluopyram (FLU) and its compound formulations, as alternatives to EB and AVM in PWD prevention. In this study, four trunk injection agents, i.e., 5% FLU microemulsion (ME), 2% AVM + 6% FLU ME, 5% EB ME, and 5% AVM emulsifiable concentrate (EC), were injected into *Pinus massoniana* trunks, and their residual dynamics over time and preventive effects on PWD were compared. Results showed that all agents were transported to various parts of the trees within 90 days post-injection, with FLU showing significantly stronger translocation compared with EB and AVM. At 660 days post-injection, the active ingredient levels of 5% FLU ME in apical branches remained significantly higher than those of the other three agents at both tested doses (30 and 60 mL). Artificial inoculation with 10,000 *Bursaphelenchus xylophilus* nematodes per tree at 90 days post-injection showed that trees injected with 5% FLU ME and 2% AVM + 6% FLU ME had nearly 100% disease prevention rates at both doses, outperforming 5% EB ME and 5% AVM EC. A second nematode inoculation at 480 days post-injection showed that 2% AVM + 6% FLU ME showed 50% efficacy, outperforming 5% EB ME (25% efficacy). These findings offer a foundation for developing alternative trunk injection strategies for future PWD management in China.

## 1. Introduction

Pine wilt disease (PWD) is a major global forest quarantine disease caused by the pine wood nematode (PWN) *Bursaphelenchus xylophilus* [1]. The disease primarily affects *Pinus* species, spreads rapidly, and is difficult to manage once it occurs. PWD is prevalent in China, Japan, Korea, the United States, Canada, Mexico, and Portugal, with China experiencing some of the most severe outbreaks, killing billions of pine trees and causing economic losses totaling hundreds of billions of yuan [2]. Therefore, more effective disease prevention strategies are urgently required.

PWD prevention and control includes plant quarantine, the removal and burning of deadwood, breeding of resistant pine trees, injecting trees with nematicides, and controlling vector beetle populations. Among these, trunk injection has become a key method owing to its simplicity, high efficiency, low environmental impact, and minimal pesticide waste with maximal uptake by the tree [3,4,5,6]. Domestic and international researchers have conducted extensive screenings of trunk injection agents for PWD prevention [7,8,9]. The nematicidal effects, along with transport and residual duration within the tree, are critical factors in selecting injection agents [6]. Emamectin benzoate (EB) and avermectin (AVM), widely used trunk injection agents, are currently considered the most effective options. However, AVM’s hydrophobic nature limits its diffusion during application [10,11]. To achieve effective control, AVM is often overused, increasing the risk of nematode resistance to pesticides [12]. Thus, there is a pressing need to identify new agents with better transport and persistence for PWD prevention.

Fluopyram (FLU), a fungicide that inhibits succinate dehydrogenase, is used to control diseases caused by fungal pathogens, e.g., *Podosphaera macularis*, *Botrytis cinerea*, and *Fusarium graminearum* [13,14,15,16]. It blocks electron transfer in succinate dehydrogenase, inhibiting mitochondrial respiration and energy production, thereby suppressing pathogen growth. This mechanism differs from that of the nerve agents EB and AVM [17,18,19,20]. Notably, Bayer CropScience has shown that FLU significantly affects various plant-parasitic nematodes, including *Meloidogyne incognita*, *Rotylenchulus reniformis*, *Meloidogyne javanica*, and *Heterodera glycines* [21,22,23,24]. In recent years, studies have shown that the half-life of fluopyram in *P. massoniana* was 346 days, with persistence lasting up to three years [25,26]. At the same time, FLU and AVM exhibit similar toxicity and reproductive inhibition [27]. Additionally, FLU’s water solubility at 20 °C is 15–16 mg/L, markedly higher than that of AVM (0.007–0.01 mg/L) [11,28,29]. These properties suggest that FLU could be a superior trunk injection agent for PWD prevention.

Previous studies from our group found that a 2% AVM + 6% FLU microemulsion (ME) exhibited significantly higher nematicidal activity, egg hatching inhibition rate, and population growth inhibition rate against *B. xylophilus* than both 5% AVM emulsifiable concentrate (EC) and 5% FLU ME [30]. However, no comparative studies have examined the pharmacokinetics and disease prevention efficacy of EB, AVM, FLU, and their combined formulations in trees. Therefore, in this study, four trunk injection agents, namely 5% FLU ME, 2% AVM + 6% FLU ME, 5% EB ME, and 5% AVM EC, were injected into forest trees to compare their transport and residue levels as well as evaluate the potential advantages of FLU and its combination formulation over EB and AVM in preventing PWD. The results provide a basis for rationally selecting trunk injection agents for PWD management and provide technical support for sustainable long-term disease control.

## 2. Results

### 2.1. Effects of Trunk Injection Agents on Pine Xylem

**Figure 1:**
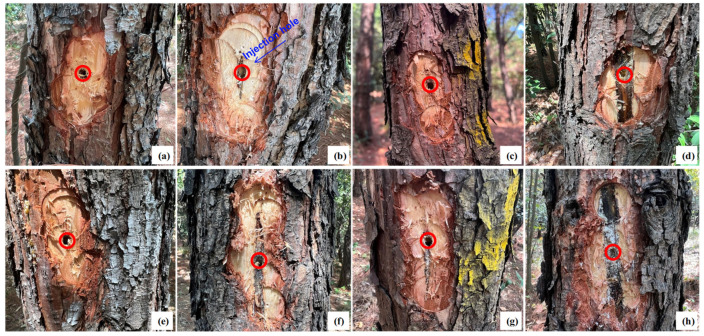
Xylem condition near the injection site at 180 days after treatment with different trunk injection agents. (**a**,**e**) Xylem responses to 30 and 60 mL of 5% EB ME. (**b**,**f**) Xylem responses to 30 and 60 mL of 5% FLU ME. (**c**,**g**) Xylem responses to 20 and 40 mL of 2% AVM + 6% FLU ME. (**d**,**h**) Xylem responses to 30 and 60 mL of 5% AVM EC. Red circles represent injection holes.

**Figure 2:**
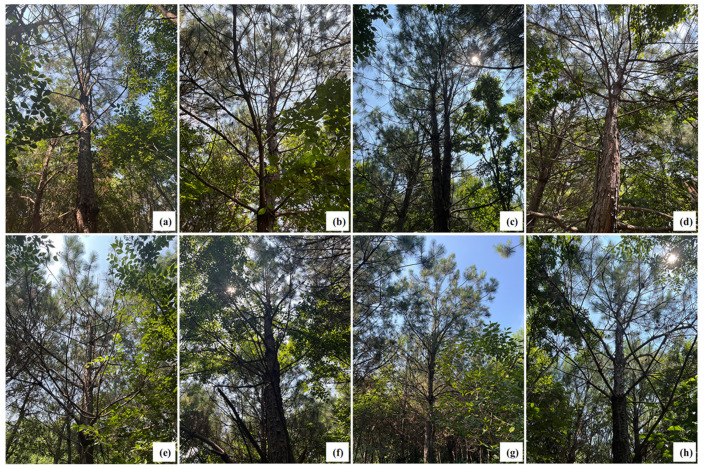
Growth condition of *P. massoniana* treated with different trunk injection agents at 180 days post-injection. (**a**,**e**) Overall vigor of 30 and 60 mL of 5% EB ME-treated *P. massoniana*. (**b**,**f**) Overall vigor of 30 mL and 60 mL of 5% FLU ME-treated *P. massoniana*. (**c**,**g**) Overall vigor of 20 and 40 mL of 2% AVM + 6% FLU ME-treated *P. massoniana*. (**d**,**h**) Overall vigor of 30 and 60 mL of 5% AVM EC-treated *P. massoniana*.

To assess the impact of the four trunk injection agents on pine tree health, xylem damage was examined at 180 days post-injection (Figure 1). No significant changes were observed in the xylem near the injection site with 30 or 60 mL of 5% EB ME (Figure 1a,e). However, high-dose treatments of 5% FLU ME (60 mL) and 2% AVM + 6% FLU ME (40 mL) caused slight blackening of the surrounding xylem, with higher damage levels than those observed with the corresponding low-dose treatments (Figure 1b,c,f,g). Injections of 30 and 60 mL of 5% AVM EB resulted in noticeable xylem darkening near the injection site (Figure 1d,h). Despite some localized tissue damage, further observation at 180 days post-injection revealed that all injected trees exhibited robust growth with healthy, emerald-green needles (Figure 2). Therefore, although certain trunk injection agents may cause localized xylem damage in *P. massoniana*, they do not significantly affect the overall health or vigor of the trees. Having assessed potential phytotoxicity at the injection site, we next examined systemic transport of the agents within the tree.

### 2.2. Transportability of Different Trunk Injection Agents in Trees

**Figure 3:**
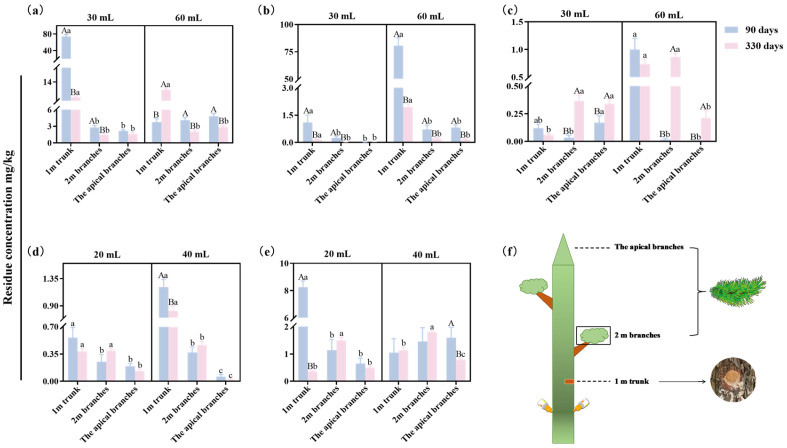
Distribution of different trunk injection agents in *P. massoniana* at 90 and 330 days post-injection. (**a**) FLU concentrations in pine trees injected with 30 and 60 mL of 5% FLU ME. (**b**) EB concentrations in pine trees injected with 30 and 60 mL of 5% EB ME. (**c**) AVM concentrations in pine trees injected with 30 and 60 mL of 5% AVM EC. (**d**) AVM concentrations in pine trees injected with 20 and 40 mL of 2% AVM + 6% FLU ME. (**e**) FLU concentrations in pine trees injected with 20 and 40 mL of 2% AVM + 6% FLU ME. (**f**) schematic diagram of the sampling site of the pine tree after injection. Results are expressed as mean ± standard deviation. The “a–c” on the error bars represent significant differences between different sampling sites under the same treatment by Duncan’s multiple range test (*p* < 0.05). The “A” and “B” on the error bars indicate significant differences between different sampling times under the same sampling site by Student’s *t*-test (*p* < 0.05).

To evaluate systemic transport, residual concentrations of the four trunk injection agents were measured at 90 days post-injection in the trunk (1 m above the injection site), branches (2 m above the injection site), and apical branches of *P. massoniana* (Figure 3). Results showed that all agents were effectively transported to the top of the trees. Among them, 5% FLU ME showed the highest apical residue levels: 2.15 and 4.84 mg/kg for the 30 and 60 mL doses, respectively, significantly exceeding those of the other agents. For the 2% AVM + 6% FLU ME treatment, the concentration of FLU in apical branches was more than 3-fold higher than that of AVM at both 20 and 40 mL doses, indicating that FLU had significantly higher transport efficiency compared with AVM in *P. massoniana*.

### 2.3. Vertical Distribution of Different Trunk Injection Agents in Pine Trees

LC-MS analysis revealed a nonuniform vertical distribution of residues across different tree tissues (Figure 3). At 90 days post-injection, high concentrations of all agents were detected in the trunk at 1 m above the injection site for both doses of 5% EB ME, 5% AVM EC, and 2% AVM + 6% FLU ME. In contrast, the concentrations in branches at 2 m above the injection site and in apical branches were similar for 5% FLU ME, 5% EB ME, and 2% AVM + 6% FLU ME (Figure 3).

At 330 days post-injection, high-dose treatments generally showed higher residue levels in all tissues compared with low-dose treatments, with significantly more pronounced differences in trunk concentrations (Figure 3). Notably, the AVM concentration in apical branches was higher for the 30 mL dose of 5% AVM EC than for the 60 mL dose (Figure 3c). Compared with 90 days post-injection, residue concentrations in the trunk at 1 m above the injection site decreased significantly for some agents by 330 days post-injection, whereas levels in branches at 2 m above the injection site and apical branches increased for some agents (Figure 3).

### 2.4. Residual Dynamics of Different Trunk-Injection Agents in Pine Trees

**Figure 4:**
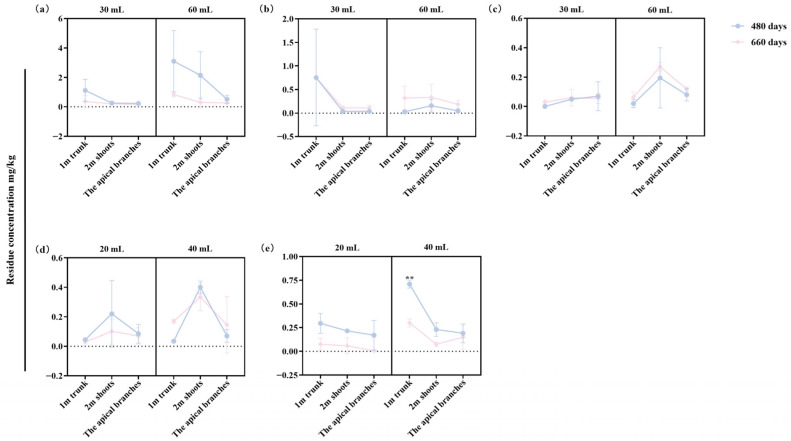
Residual levels of different trunk injection agents in pine trees at 480 and 660 days post-injection. (**a**) FLU concentrations in pine trees injected with 30 and 60 mL of 5% FLU ME. (**b**) EB concentrations in pine trees injected with 30 and 60 mL of 5% EB ME. (**c**) AVM concentrations in pine trees injected with 30 and 60 mL of 5% AVM EC. (**d**) AVM concentrations in pine trees injected with 20 and 40 mL of 2% AVM + 6% FLU ME. (**e**) FLU concentrations in pine trees injected with 20 and 40 mL of 2% AVM + 6% FLU ME. Results are expressed as mean ± standard deviation. Asterisks indicate significant differences between different sampling times under the same sampling site by Student’s *t*-test. “**” denotes *p* < 0.05.

To assess the long-term persistence of the four trunk injection agents, residue concentrations were measured at 480 and 660 days post-injection in the trunk (1 m above the injection site), branches (2 m above the injection site), and apical branches. All agents were still detectable in various parts of *P. massoniana* at both time points (Figure 4). Notably, FLU levels in the apical branches remained higher for both 30 and 60 mL doses of 5% FLU ME than for the corresponding high- and low-doses of the other three agents. At 660 days post-injection, the concentrations of active ingredients in trees injected with either dose of 5% EB ME or 5% AVM EC were similar to or even higher than those observed at 480 days post-injection.

### 2.5. Efficiency of Different Trunk-Injection Agents Against PWD

To determine the protective efficacy of the four trunk injection agents against PWD, *B. xylophilus* was artificially inoculated into trees at 90 days post-injection in June 2022, with uninjected, *B. xylophilus*–inoculated trees serving as the CK. As shown in Table 1, the disease severity index (DSI) of pine trees in the CK reached 8.33 and 100 at 20 and 270 days post-inoculation (dpi), respectively. In contrast, trees injected with either dose of 5% FLU ME or 20 mL of 2% AVM + 6% FLU ME showed complete protection (100% efficacy) at 270 dpi. Trees injected with 40 mL of 2% AVM + 6% FLU ME had a DSI of 8.33 and a prevention efficacy of 91.7%, both of which were better than those of trees treated with 5% EB ME or 5% AVM EC. Notably, the high-dose 5% AVM EC treatment was less effective than the corresponding low-dose treatment. These results indicate that 5% FLU ME and 2% AVM + 6% FLU ME offer the potential to replace traditional EB and AVM formulations for PWD prevention.

To further evaluate the long-term efficacy of 5% FLU ME and 2% AVM + 6% FLU ME against PWD, a second artificial inoculation with *B. xylophilus* was performed at 480 days post-injection (approximately 1.5 years). At 60 dpi, trees injected with either dose of 5% FLU ME and 20 mL of 2% AVM + 6% FLU ME began to display susceptibility, with DSI of 16.67, 8.33, and 8.33, respectively (Table 2). By 220 dpi, trees injected with either dose of 2% AVM + 6% FLU ME had a DSI of 33.33 and a prevention efficacy of 50%. In comparison, the DSI for trees injected with 5% FLU ME was 50, with a prevention efficacy of 25% (Table 2). These results suggest that 2% AVM + 6% FLU ME provides more durable disease prevention compared with 5% FLU ME alone.

## 3. Discussion

The effectiveness of EB and AVM in preventing PWD has been well established [6,7]. FLU, a novel, water-soluble nematicide, was first reported in 2020 to exhibit nematicidal activity comparable to that of AVM, with an LC_50_ value of only 0.945 mg/L [27]. Previous trials in our laboratory confirmed that 5% FLU ME and 2% AVM + 6% FLU ME show significantly stronger nematicidal activity than that of 5% AVM EC [30]. However, no studies have systematically compared the pharmacokinetics of EB, AVM, FLU, and their compound formulations in pine trees, nor have their relative field-level efficacies in PWD prevention been determined. The present study addresses these critical gaps.

Although trunk injection enables efficient systemic delivery of pesticides, it can also lead to phytotoxic effects due to interactions between the chemical agents and the tree’s physiological systems [31,32,33]. In this study, the ME formulations of 5% FLU ME, 5% EB ME, and 2% AVM + 6% FLU ME caused minimal tissue damage at all tested doses. In contrast, 5% AVM EC (formulated as an emulsifiable concentrate) induced noticeable xylem damage around the injection site, regardless of dose. This aligns with previous reports indicating that emulsifiable concentrates, often containing aromatic hydrocarbons, such as benzene or xylene, may enhance pesticide penetration but also elevate phytotoxic risk [6,34]. Nevertheless, the present study suggests that overall tree growth remained unaffected by any of the four trunk injection agents, indicating their general safety in field applications.

The persistence and efficacy of PWD control agents are closely related to their transport kinetics and residual dissipation dynamics within the host tree. In the trunk injection system, radial transport of the agent in the xylem tissue is mainly driven by transpiration, making water solubility a key determinant of systemic distribution [35]. AVM’s low water solubility (0.01 mg/L at 21 °C) may lead to migration hysteresis effects during longitudinal xylem transport [11,29]. Residue dynamics analysis has shown that AVM’s digestion half-life (DT_50_) is 75 days in jujube trees, with a 67% decline from the peak concentration at 120 days post-injection due to residue decay [36]. In contrast, FLU demonstrated a DT_50_ of up to 365 days in *P. massoniana* xylem tissues, indicating stronger residual efficacy [25]. In our study, all four trunk injection agents exhibited systemic transport properties in pine trees, but FLU showed superior mobility relative to EB and AVM at 90 and 330 days post-injection. At 660 days post-injection, the concentrations of active ingredients in trees injected with either dose of 5% EB ME or 5% AVM EC were higher than or close to the levels at 480 days post-injection. We speculate that this observation is likely closely related to the seasonal physiological activity of trees and the physicochemical properties of the pesticides. Notably, the residual concentrations of 5% FLU ME in the apical branches remained higher than those of the other agents even at 660 days post-injection. These findings confirm FLU’s dual advantage in terms of systemic transport and residual persistence in pine trees.

Consistent with these pharmacokinetic profiles, two rounds of artificial inoculation with *B. xylophilus* showed that 2% AVM + 6% FLU ME provided significantly longer-lasting protection relative to the other agents. Several months after the second inoculation, disease prevention efficiency remained at 50%, compared with 25% for 5% FLU ME. Previous studies have reported that in *P. massoniana* (diameter at breast height: ~10–13 cm; height: ~9–11 m), injection of 30 mL of 5% EB soluble liquid or 35 mL of 5% AVM EC, followed by artificial inoculation with 10,000 nematodes per tree at 450 d post-injection, resulted in DSI of 16.67 and 8.3, respectively, at 45 d post-inoculation [7]. By contrast, in the present study, *P. massoniana* injected with 5% EB ME or 5% AVM EC and inoculated with an equivalent number of nematodes at 90 d post-injection exhibited higher DSI at 270 d post-inoculation than those reported previously. We speculate that this discrepancy may be attributable to differences in the adjuvant components used in the tested formulations, which could influence the persistence of the active ingredients within pine tissues and thereby attenuate protective efficacy during long-term observation. In addition, it should be noted that the sample size used to evaluate the transport ability, residual persistence, and protective efficacy of the different agents in *P. massoniana* was relatively limited, which may have constrained the statistical power and the generalizability of the findings. Future studies should therefore incorporate larger sample sizes and increased biological replication to enable more rigorous statistical analyses and significance testing, thereby enhancing the robustness and broader applicability of the results and providing a more solid evidence base for the scientifically informed application of these agents in practical forestry management. Taken together, this study’s results showed that, under the tested conditions, FLU and its compound formulation have substantial advantages over EB and AVM in terms of persistence and protective efficacy. These properties support their potential as novel control agents for PWD.

## 4. Materials and Methods

### 4.1. PWN Material and Cultivation

The PWN *B. xylophilus* isolate AMA3 (Anhui Province, China) used in this study was obtained from naturally infected *P. massoniana* and is currently maintained in the Forest Protection Laboratory, Nanjing Forestry University [37]. Experimental nematodes were cultured on mycelial mats of *Botrytis cinerea* grown on potato dextrose agar at 25 °C for 1 week [38].

### 4.2. Test Agent

5% FLU ME, 2% AVM + 6% FLU ME, 5% EB ME, and 5% AVM EC were provided by Esenj Biotechnology Co., Ltd. (Hangzhou, China).

### 4.3. Test Plots

The test site was located in Lutang Industrial District, Jurong City, Jiangsu Province (119°15′ E, 32°05′ N). The site has a subtropical monsoon climate with four distinct seasons, warm and humid conditions, abundant rainfall, and mean winter temperatures above 0 °C. Healthy 10-year-old *P. massoniana* trees were selected (canopy density: >70%) with a diameter at breast height: ~10–13 cm; height: ~7–9 m. The trees were evenly distributed, untreated with pesticides, and free of PWD infection.

### 4.4. Effects of Different Trunk-Injection Agents on the Growth of Pine Trees

**Figure 5:**
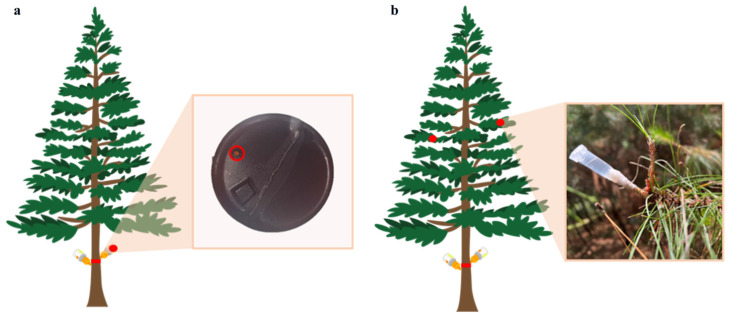
Schematic of injection method and artificial inoculation of *B. xylophilus*. (**a**) Self-flowing, double-sided injection (red circle indicates the bottom hole). (**b**) Branch cutting and casing method used for *B. xylophilus* inoculation.

To assess the potential adverse effects of the four agents on *P. massoniana* growth, a self-flowing trunk injection method was applied in March 2022. Holes were drilled on both sides of the trunk (drill at a 45° angle to a depth of 4–5 cm), injection bottles were inserted, and the bottom of each bottle was punctured (Figure 5a). Following the protocol of Xiang et al. [7], trunk injection of 30 mL of a 5% EB soluble liquid in *P. massoniana* (diameter at breast height: ~10–13 cm; height: ~9–11 m) resulted in a disease severity index of 83.3 at 640 days post-injection. Each agent was injected at two concentrations. Injection doses were as follows: 30 and 60 mL for 5% FLU, 5% EB ME, and 5% AVM EC; and 20 and 40 mL for 2% AVM + 6% FLU ME. Each treatment was injected into five *P. massoniana* trees. The xylem near the injection site and overall tree vigor were examined at 180 days post-injection. Two trees per dose were evaluated for each agent.

### 4.5. Distribution and Residue Dynamics of Different Trunk-Injection Agents in Trees

To evaluate the transport and persistence of four trunk injection agents in *P. massoniana,* residue analyses were performed on trees treated as described in Section 4.4 at 90, 330, 480, and 660 days post-injection. Samples were taken from three positions within each tree: trunks at 1 m above the injection point, shoots at 2 m, and apical branches. For each treatment, two independent trees were selected, and at each time point, samples were taken from all three positions on both trees. Each sampling point was sampled once, resulting in six samples per treatment and a total of 48 samples per time point.

### 4.6. Processing and Extraction of Tree Samples

Shoot samples collected 2 m above the injection point and from the apical branches were debarked and cut into small pieces. In parallel, trunk samples were obtained 1 m above the injection point by removing the bark with an electric drill and drilling to a depth of 4–5 cm. All samples were air-dried and pulverized before being sent to Qingdao Stander Standard Testing Co., Ltd. for analysis. Following Deng et al. [39], 10 mL of 50% acetonitrile was added to each homogenized sample (5 g) using a vortex oscillator (Shanghai Qingpu Huxi Instrument Factory, Shanghai, China). Samples were then sonicated for 15 min in an ultrasonic bath (KQ-500E, Kunshan, China) before centrifugation at 6000 rpm for 5 min. Supernatants were purified using C18 columns, eluted with 3 mL of acetonitrile, dried under nitrogen, reconstituted with 1 mL of the mobile phase, and filtered using 0.22 μm organic membranes for liquid chromatography-mass spectrometry (LC-MS) analysis.

### 4.7. LC-MS Analysis

According to the GB/T 20769-2008 method [40], agent residue concentrations in the trunk (1 m above the injection point), shoots (2 m above the injection point), and apical branches were analyzed via LC-MS at 90, 330, 480, and 660 dpi. An Agilent C18 column (2.1 mm × 100 mm, 1.8 μm) was used at 35 °C with a mobile phase ratio of 5 mmol/L ammonium acetate/acetonitrile (90:10), a flow rate of 0.3 mL/min, an electrospray ionization source, positive ion scanning, and multiple reaction monitoring scanning. Injection volume was 5 μL. The detection limit ranges from 0.01 μg/kg to 0.606 mg/kg. Residue concentrations in each sample were estimated as follows:

(1) $$W=\frac{\left(C\;-\;C_{0}\right)\;\times \;V\;\times \;N}{m}$$

where W denotes the concentration of the target analyte in the sample (μg/kg), C represents the concentration of the target analyte in the sample extract (μg/L), C_0_ is the concentration of the target analyte in the blank control (μg/L), V is the extract volume (mL), N is the dilution factor, and m is the sample mass (mL).

### 4.8. Efficacy of Different Trunk-Injection Agents Against PWD

To assess the efficacy of the four agents in preventing PWD, the remaining three treated *P. massoniana* (refer to Section 2.3) were inoculated with *B. xylophilus* in June 2022 (Figure 5b). Specifically, at 90 days post-injection, the apical segments of selected branches were excised, and 5 mL centrifuge tubes were fitted onto the cut ends. A nematode suspension was then introduced into each tube. Two lateral branches per tree were inoculated, with 5000 nematodes per branch (10,000 nematodes per tree in total). Disease progression was monitored, and uninjected, *B. xylophilus*–inoculated trees served as the control group (CK). Three trees were inoculated per treatment.

To further evaluate 5% FLU ME and 2% AVM + 6% FLU ME efficacy against PWD, a second inoculation with *B. xylophilus* (two side branches were inoculated per tree, with 5000 nematodes per side branch) was performed in July 2023 on trees treated in June 2022, and their disease status was monitored continuously. Three trees were inoculated per treatment.

Disease symptoms were recorded regularly. The disease grading and disease index followed Zhang et al. [6], with slight modifications: grade 0: normal; grade 1: <10% needle discoloration; grade 2: 10–50% needle discoloration; grade 3: >50% needle discoloration; grade 4: full needle discoloration or death. Branches were collected from each dead tree, separated using the Baermann funnel method, and tested for nematodes to confirm PWD-induced mortality. The disease severity index and PWD control efficacy were calculated as follows:

(2) $$\mathrm{Disea}(1)se\;\mathrm{severity}\;\mathrm{index}\;\left(\mathrm{DSI}\right)=\frac{\sum \mathrm{Number}\;\mathrm{of}\;\mathrm{disease}\;\mathrm{plants}\;\times \;\mathrm{symptom}\;\mathrm{stage}}{\mathrm{Total}\;\mathrm{number}\;\mathrm{of}\;\mathrm{plants}\;\times \;\mathrm{highest}\;\mathrm{symptom}\;\mathrm{stage}}\;\times \;100\;$$

(3) $$\begin{array}{c} \mathrm{Efficac}(2)y\;\mathrm{against}\;\mathrm{pine}\;\mathrm{wilt}\;\mathrm{disease}\;\left(\%\right)=\\ \frac{\mathrm{DSI}\;\mathrm{in}\;\mathrm{the}\;\mathrm{control}\;\mathrm{group}\;-\;\mathrm{DSI}\;\mathrm{in}\;\mathrm{the}\;\mathrm{treament}\;\mathrm{group}}{\mathrm{DSI}\;\mathrm{in}\;\mathrm{the}\;(3)\mathrm{control}\;\mathrm{group}}\;\times \;100\; \end{array}$$

## 5. Conclusions

Among the four evaluated trunk injection agents, FLU exhibited superior systemic transport in pine trees, with consistently higher residual FLU concentrations in apical branches compared with EB and AVM. Two consecutive *B. xylophilus* inoculation tests confirmed that disease prevention efficacy followed the order 2% AVM + 6% FLU ME > 5% FLU ME > 5% EB ME > 5% AVM EC. These findings suggest that FLU and its compound formulations are promising alternatives to traditional EB and AVM products, offering a strategic solution to potential PWD resistance problems and expanding the range of effective agents available for long-term PWD control.

## Figures and Tables

**Table 1:** Disease prevention efficacy of *B. xylophilus* inoculation of different trunk injection agents at 90 days (3 months) post-injection.

Treatment	Injection Volume (mL)	Disease Severity Index (DSI)				Efficacy Against PWD
		20 dpi	40 dpi	70 dpi	270 dpi	
CK	/	8.33	58.33	83.33	100	/
5% FLU	30	0	0	0	0	100
ME	60	0	0	0	0	100
5% EB	30	0	16.67	50	66.67	33.33
ME	60	0	0	0	25	75
5% AVM	30	0	0	0	16.67	83.33
EC	60	0	8.33	16.67	41.67	58.33
2% AVM + 6% FLU ME	20	0	0	0	0	100
	40	0	0	0	8.33	91.67

Note: dpi denotes days post inoculation.

**Table 2:** Disease prevention efficacy of FLU and its compound formulation against *B. xylophilus* following a second inoculation at 480 days (approximately 1.5 years) post-injection.

Treatment	Injection Volume (mL)	Disease Severity Index (DSI)			Efficacy Against PWD
		60 dpi	150 dpi	220 dpi	
5% FLU ME	30	16.67	33.33	50.00	25
	60	8.33	33.33	50.00	25
2% AVM + 6% FLU ME	20	8.33	16.67	33.33	50
	40	8.33	8.33	33.33	50

Note: dpi denotes days post inoculation.

## Data Availability

The original contributions presented in this study are included in the article. Further inquiries can be directed to the corresponding author.
